# Transcriptomes of *Frankia *sp. strain CcI3 in growth transitions

**DOI:** 10.1186/1471-2180-11-192

**Published:** 2011-08-25

**Authors:** Derek M Bickhart, David R Benson

**Affiliations:** 1 Department of Molecular and Cell Biology, U-3125, University of Connecticut, Storrs, CT, USA; 2 Bovine Functional Genomics Laboratory, USDA-ARS, Building 200, Room 124B, BARC-East, Beltsville, MD, 20705, USA

## Abstract

**Background:**

*Frankia *sp. strains are actinobacteria that form N_2_-fixing root nodules on angiosperms. Several reference genome sequences are available enabling transcriptome studies in *Frankia *sp. Genomes from *Frankia *sp. strains differ markedly in size, a consequence proposed to be associated with a high number of indigenous transposases, more than 200 of which are found in *Frankia *sp. strain CcI3 used in this study. Because *Frankia *exhibits a high degree of cell heterogeneity as a consequence of its mycelial growth pattern, its transcriptome is likely to be quite sensitive to culture age. This study focuses on the behavior of the *Frankia *sp. strain CcI3 transcriptome as a function of nitrogen source and culture age.

**Results:**

To study global transcription in *Frankia *sp. CcI3 grown under different conditions, complete transcriptomes were determined using high throughput RNA deep sequencing. Samples varied by time (five days vs. three days) and by culture conditions (NH_4_^+ ^added vs. N_2 _fixing). Assembly of millions of reads revealed more diversity of gene expression between five-day and three-day old cultures than between three day old cultures differing in nitrogen sources. Heat map analysis organized genes into groups that were expressed or repressed under the various conditions compared to median expression values. Twenty-one SNPs common to all three transcriptome samples were detected indicating culture heterogeneity in this slow-growing organism. Significantly higher expression of transposase ORFs was found in the five-day and N_2_-fixing cultures, suggesting that N starvation and culture aging provide conditions for on-going genome modification. Transposases have previously been proposed to participate in the creating the large number of gene duplication or deletion in host strains. Subsequent RT-qPCR experiments confirmed predicted elevated transposase expression levels indicated by the mRNA-seq data.

**Conclusions:**

The overall pattern of gene expression in aging cultures of CcI3 suggests significant cell heterogeneity even during normal growth on ammonia. The detection of abundant transcription of *nif *(nitrogen fixation) genes likely reflects the presence of anaerobic, N-depleted microsites in the growing mycelium of the culture, and the presence of significantly elevated transposase transcription during starvation indicates the continuing evolution of the *Frankia *sp. strain CcI3 genome, even in culture, especially under stressed conditions. These studies also sound a cautionary note when comparing the transcriptomes of *Frankia *grown in root nodules, where cell heterogeneity would be expected to be quite high.

## Background

Studies on actinorhizal symbioses have benefitted greatly from several genome sequences of the actinobacterial symbiont *Frankia *sp. strains. Such strains induce root nodules and fix N_2 _in a broad array of plants [1]. The smallest frankial genome finished to date is that of *Frankia *sp. HFPCcI3 (CcI3) that infects plants of the family Casuarinaceae; it is about 5.4 Mbp in size and encodes 4499 CDS [2]. A striking feature of the CcI3 genome is the presence of over 200 transposase genes or gene remnants that may play, or have played, a role in genome plasticity [3]. In addition, relative to other *Frankia *sp. genomes that have been sequenced, CcI3 contains few gene duplicates [2]. Comparative genome studies suggest that evolution has favored gene deletion rather than duplication in this strain, perhaps as an outcome of its symbiotic focus on a single, geographically limited group of plants in the Casuarinaceae [2].

Transcriptome sequencing of bacterial genomes has yielded surprising complexity (for a review see [4]). Such studies have shown differential cistron transcription within operons [5], small regulatory RNA transcripts [6-9] and numerous riboswitch controlled transcripts [10,11]. Significant transcriptional heterogeneity has also been found in single cultures that has been ascribed to subpopulations within an otherwise synchronized bacterial population [12]. High throughput RNA-seq methods provide a tool for transcript quantification with a much higher dynamic range than that provided by microarray studies by relying on direct comparison of transcript abundance for assessing differential expression [13].

*Frankia *transcriptome studies have the potential to reveal common genes and pathways active in, or essential to, symbiosis and free-living growth. A first step to resolving symbiotic-specific expression is to gain insight into transcriptional behavior and variability in axenic culture. This work helps address the issue of cultural heterogeneity that will likely be exacerbated by physiological heterogeneity in symbiosis. A previous transcriptome study has been done using whole-genome microarrays in *Alnus *and *Myrica *root nodules using cultured *Frankia alni *strain ACN14a as a reference [14]. In that study, relatively few surprises were encountered and the overall transcription profile was similar in both nodule types. We focus here on an approach using transcriptome deep sequencing of cultured *Frankia *strain CcI3 grown under different conditions, and the analysis of subsequent data to provide insight into the global expression that may impinge on physiology and genome stability in *Frankia *strains.

## Results and Discussion

### Culture characteristics and experimental design

As a consequence of its filamentous growth habit, *Frankia *sp. strain CcI3 grows from hyphal tips with an initial doubling time of about 18 hrs that subsequently slows to more linear growth [15]. As tips extend, cells left behind are physiologically in stationary phase and eventually senesce. Thus, even young cultures (defined here as three days old) have a degree of physiological heterogeneity that increases as cultures age [16]. This heterogeneity must be taken into account in interpreting global transcriptome analyses.

Several factors in our sampling and library creation may influence a transcriptome analysis. Single *Frankia *cultures were used in preparing RNA libraries for each sample prior to sequencing. In addition, each sample was run on the Illumina GA IIx sequencer without technical replicates. While technical and biological replicates would have eliminated two potential sources of variability in the results of this experiment, several studies have suggested that both types of variability are unlikely to influence end results [13,17], while other studies have found significant variation among replicate samples [18,19]. Such effects may only influence low RPKM value genes [20] but, as with many such studies, our results must be viewed in the light of many potential variables.

### RNA sample quality and features

RNA preparations used for making dscDNA libraries for Illumina sequencing had 260/280 ratios greater than 2.0 and greater than 400 to 950 ng per μl. PCR amplification using primers for the *glnA *gene failed to yield an amplicon from RNA preparations indicating very low, if any, DNA contamination. In addition, an RT-PCR assay revealed no detectable DNA within total RNA samples prepared in a separate experiment, confirming that the RNA extraction technique can apply to sensitive RNA based experiments that use strain CcI3.

Transcriptome sequencing done using 5dNH4 CcI3 cells yielded about six million reads, three million of which could be mapped to the *Frankia *sp. CcI3 genome (Table 1). Almost 51% of the mapped reads were from rRNA or tRNA (Table 1). An updated base-calling algorithm (RTA v. 1.6) yielded substantially higher reads for samples from 3dNH4 and 3dN2 cultures. About 26 million reads were obtained for the latter samples, with about 16 million mapped reads in each (Table 1). Non-coding RNAs represented a greater proportion of mapped reads in these two samples, comprising nearly 80% of the total.

**Table 1:** Dataset statistics

	5dNH4 (#ORFs/#Reads^ǂ^)	3dNH4 (#ORFs/#Reads^ǂ^)	3dN2 (#ORFs/#Reads^ǂ^)
**rRNA/tRNA**	**65/1,401,120**	**65/12,799,049**	**64/13,524,803**
**mRNA**	**4,491/1,322,139**	**4,544/2,813,063**	**4544/2,945,205**
hypothetical	1,355/307,027	1,363/547,196	1,363/634,786
pseudogenes	49/8,882	49/31,566	49/44,989
transposases	135/24,528	137/62,484	137/87,928
phage proteins	26/12564	26/17,292	26/25,218
CRISPRs	9/6,553	9/8,926	9/12,702

^ǂ ^Includes reads that mapped ambiguously. Ambiguous reads were only counted once.

Even after ribosomal RNA depletion, non-coding sequences formed the majority of reads in all samples with the greatest reduction seen in the 5dNH4 sample (Table 1). This relative amount of rRNA could be related to the reduction of rRNA in older cultures, as observed in stationary and death phase cultures of *E. coli *[21]. On the other hand, given the concentration dependence of the rRNA depletion method used in preparing the mRNA-seq libraries, a decrease in the proportion of rRNA in the five-day time point could have resulted from more efficient depletion. Incomplete depletion of rRNA populations is similar to what is observed in other studies and is related to the sheer abundance of such sequences [22].

The number of coding RNA reads was similar among all three samples although the read length for the 3dNH4 and 3dN2 samples was 76 versus 34 for 5dNH4. All of the pseudogenes present in the CcI3 genome had transcripts in at least two of the three genomes (Table 1). Pseudogene transcription is presently not believed be a rare event [23], though many pseudogenes identified in a bacterial genome may simply be misannotated ORFS.

### Functional Pathways

The 100 genes with the highest RPKM value in each condition, omitting ribosomal RNAs, are listed in Table 2. The number of hypothetical genes in this group range from 29 in the 3dNH4 cells to 39 in the 3dN2 cells to 43 in the 5dNH4 cells. Older cultures had more transcripts associated with tRNAs, transposases, CRISPR elements, integrases and hypothetical proteins than did younger cultures. Indeed, had they been included in the list, 18 of the 46 tRNA genes in CcI3 would have been in the top 100 most abundant transcript populations in 5dNH4 cells whereas no tRNAs were found in the top 100 transcripts in 3dN2 or 3dNH4 cell populations. The picture painted by the abundance of such transcripts is one of cells starved for essential metabolites such as amino acids, as expected in aging cells. In addition, enzymes involved in solving oxidative damage (e.g. protein-methionine-S-oxide reductase) were also more abundant in the older culture. Conversely, enzymes involved in catabolism (eg. alcohol dehydrogenase) were more frequently represented in the two younger cultures.

**Table 2:** The top 100 highly expressed coding ORFs predicted by RPKM values

3dNH4^1^	Locus tag	RPKM^2^	3dN2	Locus tag	RPKM	5dNH4	Locus tag	RPKM
**heat shock protein Hsp20**	**Francci3_1179**	**10755**	**heat shock protein Hsp20**	**Francci3_1179**	**3553**	hypothetical protein	Francci3_1017	4967
**aldehyde dehydrogenase**	**Francci3_2944**	**7165**	**aldehyde dehydrogenase**	**Francci3_2944**	**3152**	**heat shock protein Hsp20**	**Francci3_1179**	**2077**
**chaperonin GroEL**	**Francci3_4398**	**5923**	**hypothetical protein**	**Francci3_1545**	**2327**	**hypothetical protein**	**Francci3_3999**	**1926**
cold-shock DNA-binding	Francci3_0260	5495	**transposase IS66**	**Francci3_1864**	**2261**	**transposase IS66**	**Francci3_1864**	**1801**
OsmC-like protein	Francci3_4465	5490	hypothetical protein	Francci3_2178	1993	polysaccharide deacetylase	Francci3_0165	1616
co-chaperonin GroES	Francci3_0632	5362	response regulator receiver	Francci3_0120	1823	hypothetical protein	Francci3_2101	1596
Hemerythrin HHE cation	Francci3_1066	4392	Hemerythrin HHE cation	Francci3_1066	1807	phage integrase	Francci3_4274	1451
**hypothetical protein**	**Francci3_1545**	**4225**	hypothetical protein	Francci3_1936	1789	Radical SAM	Francci3_1753	1392
NAD/NADP transhydrogenase	Francci3_2947	3226	OsmC-like protein	Francci3_4465	1777	hypothetical protein	Francci3_2241	1333
**UspA**	**Francci3_2760**	**3221**	**hypothetical protein**	**Francci3_3999**	**1614**	hypothetical protein	Francci3_2890	1265
hypothetical protein	Francci3_3494	3190	cold-shock DNA-binding	Francci3_0260	1592	phosphoribosyl-ATPphosphatase	Francci3_4317	1245
hypothetical protein	Francci3_2178	3071	sigma 54 modulation	Francci3_0764	1574	hypothetical protein	Francci3_0159	1184
sigma 54 modulation protein	Francci3_0764	3004	cold-shock DNA-binding	Francci3_4469	1458	ribonucleaseHII	Francci3_3588	1161
cold-shock DNA-binding	Francci3_4469	2949	putative DNA-binding	Francci3_1949	1392	GDP-mannose 4,6-dehydratase	Francci3_1307	1134
Alcohol dehydrogenase	Francci3_2945	2916	LuxR family regulator	Francci3_0765	1361	hypothetical protein	Francci3_4023	1122
putative Lsr2-like protein	Francci3_3498	2659	**chaperoninGroEL**	**Francci3_4398**	**1199**	major facilitator superfamily	Francci3_2289	1122
hypothetical protein	Francci3_1936	2577	hypothetical protein	Francci3_4123	1176	RNA-directed DNA polymerase	Francci3_2318	1088
hypothetical protein	Francci3_2270	2529	hypothetical protein	Francci3_3494	1175	methionine-S-oxide reductase	Francci3_2268	1071
thioredoxin-related	Francci3_0447	2355	hypothetical protein	Francci3_2269	1174	HypA	Francci3_1937	1047
**SsgA**	**Francci3_3418**	**2154**	transcriptional regulator	Francci3_4255	1167	acyltransferase 3	Francci3_2337	987
luciferase-like	Francci3_2761	2117	co-chaperoninGroES	Francci3_0632	1150	hypothetical protein	Francci3_3302	982
molecular chaperone DnaK	Francci3_4352	2036	hypothetical protein	Francci3_2442	1117	Serine acetyltransferase-like	Francci3_3842	970
globin	Francci3_2581	1935	**SsgA**	**Francci3_3418**	**1043**	**hypothetical protein**	**Francci3_0227**	**970**
LuxR family regulator	Francci3_0765	1934	SecE subunit	Francci3_0567	1037	hypothetical protein	Francci3_1719	965
thioredoxin reductase	Francci3_4536	1913	putative Lsr2-like protein	Francci3_3498	1022	hypothetical protein	Francci3_0238	957
Rhodanese-like	Francci3_0449	1881	PEP phosphomutase	Francci3_1533	1005	hypothetical protein	Francci3_2200	947
carbonic anhydrase	Francci3_0708	1859	hypothetical protein	Francci3_2270	973	hypothetical protein	Francci3_1831	945
**superfamily MFS_1**	**Francci3_2752**	**1811**	chaperone hypC/hupF	Francci3_1946	954	serine/threonine kinase	Francci3_4051	938
hypothetical protein	Francci3_3250	1807	transposase, IS4	Francci3_3990	953	signal transduction kinase	Francci3_0085	938
exodeoxyribonuclease III	Francci3_1180	1754	thioredoxin-related	Francci3_0447	951	hypothetical protein	Francci3_4019	922
PEP phosphomutase	Francci3_1533	1742	ATP synthase F0	Francci3_3713	928	hypothetical protein	Francci3_0396	914
STAS (anti-σ factor antagonist)	Francci3_0441	1728	mannose 4,6-dehydratase	Francci3_1053	921	CRISPR-associated protein	Francci3_0021	899
hypothetical protein	Francci3_1935	1687	phage integrase	Francci3_4338	919	hypothetical protein	Francci3_0038	899
sigma 38	Francci3_3505	1673	protein of unknown function	Francci3_3347	892	Recombinase	Francci3_3989	898
**hypothetical protein**	**Francci3_0227**	**1665**	transposase, IS4	Francci3_0391	878	aldo/keto reductase	Francci3_3416	890
hypothetical protein	Francci3_1615	1634	**major facilitator MFS_1**	**Francci3_2752**	**865**	transposase, IS4	Francci3_1873	875
hypothetical protein	Francci3_2943	1629	NAD/NADP transhydrogenase	Francci3_2947	863	Excisionase/Xis, DNA-binding	Francci3_0405	875
hypothetical protein	Francci3_0054	1629	hypothetical protein	Francci3_4084	855	transposase, IS4	Francci3_0151	874
**transposase IS66**	**Francci3_1864**	**1625**	*hypothetical protein*	*Francci3_2380*	*839*	CRISPR-associated protein	Francci3_0020	869
transcriptional regulator, CarD	Francci3_4255	1596	hypothetical protein	Francci3_4114	821	*CRISPR-associated protein*	*Francci3_3345*	*863*
alanine dehydrogenase/PNT-like	Francci3_2946	1532	Alcohol dehydrogenase	Francci3_2945	796	glycosyl transferase	Francci3_3318	859
serine phosphatase	Francci3_3249	1453	hypothetical protein	Francci3_3791	782	metallophosphoesterase	Francci3_1990	839
chaperonin GroEL	Francci3_0633	1439	acyl-CoA dehydrogenase	Francci3_1000	781	hypothetical protein	Francci3_3339	837
hypothetical protein	Francci3_0949	1437	*transcriptional regulator*	*Francci3_3081*	*780*	*transcriptional regulator*	*Francci3_3081*	*834*
transcription factor WhiB	Francci3_3759	1430	*hypothetical protein*	*Francci3_0037*	*779*	hypothetical protein	Francci3_3317	826
fatty acid desaturase, type 2	Francci3_0307	1430	Amino acid adenylation	Francci3_2461	777	hypothetical protein	Francci3_4072	824
STAS	Francci3_4302	1405	hypothetical protein	Francci3_1615	775	transcriptional regulator	Francci3_0908	816
Heavy metal transportprotein	Francci3_0489	1368	hypothetical protein	Francci3_2179	775	hypothetical protein	Francci3_4129	809
sigma-24	Francci3_3768	1353	hypothetical protein	Francci3_1534	773	transposase, IS4	Francci3_4227	803
transcriptional regulator, TetR	Francci3_2758	1349	hypothetical protein	Francci3_2329	767	**Antibiotic biosynthesis**	**Francci3_0875**	**800**
hypothetical protein	Francci3_3417	1343	carbonic anhydrase	Francci3_0708	764	hypothetical protein	Francci3_3336	796
SecE subunit	Francci3_0567	1339	transcription factor WhiB	Francci3_3759	751	*hypothetical protein *	*Francci3_2440*	*781*
Excisionase/Xis, DNA-binding	Francci3_0099	1327	**UspA**	**Francci3_2760**	**747**	*hypothetical protein*	*Francci3_4509*	*778*
hypothetical protein	Francci3_3791	1315	exodeoxyribonuclease III	Francci3_1180	747	putative copper resistance	Francci3_2497	771
ATP synthase F0, A subunit	Francci3_3713	1263	hypothetical protein	Francci3_1832	737	transcriptional regulator	Francci3_0210	765
30S ribosomal proteinS1	Francci3_1057	1256	protein of unknown function	Francci3_2628	714	hypothetical protein	Francci3_1090	764
heat shock protein Hsp20	Francci3_2174	1241	hypothetical protein	Francci3_4509	714	hypothetical protein	Francci3_4156	760
NAD(P) transhydrogenase, beta	Francci3_2948	1231	hypothetical protein	Francci3_1650	709	RNA-binding S4	Francci3_3479	747
putative transcriptional regulator	Francci3_1674	1218	STAS	Francci3_0441	701	**hypothetical protein**	**Francci3_1545**	**746**
protein of unknown function	Francci3_0450	1215	molecularchaperoneDnaK	Francci3_4352	694	hypothetical protein	Francci3_3238	746
Alcohol dehydrogenase	Francci3_1544	1206	hypothetical protein	Francci3_0159	693	hypothetical protein	Francci3_3301	737
putative DNA-binding protein	Francci3_1949	1203	acyl transferase region	Francci3_0991	691	hypothetical protein	Francci3_1985	724
glutaredoxin 2	Francci3_0483	1202	regulatory protein GntR	Francci3_3218	690	*Rhodanese-like *	*Francci3_2753*	*721*
translation elongation factor Tu	Francci3_0580	1179	CRISPR-associated protein	Francci3_3346	680	Thiolase	Francci3_2502	718
thioredoxin	Francci3_4537	1165	hypothetical protein	Francci3_1874	678	response regulator receiver	Francci3_0120	715
cytochrome P450	Francci3_4464	1164	hypothetical protein	Francci3_1935	672	hypothetical protein	Francci3_0498	705
hypothetical protein	Francci3_2582	1156	IS630 family transposase	Francci3_1872	670	DNApolymeraseIIIsubunitalpha	Francci3_4168	703
hypothetical protein	Francci3_1534	1106	globin	Francci3_2581	663	*hypothetical protein*	*Francci3_0037*	*693*
protein of unknown function	Francci3_1406	1054	hypothetical protein	Francci3_4127	657	hypothetical protein	Francci3_3241	684
Vesicle-fusing ATPase	Francci3_2630	1041	thioredoxin	Francci3_4537	653	30SribosomalproteinS6	Francci3_4522	683
HesB/YadR/YfhF	Francci3_3121	1032	hypothetical protein	Francci3_0066	644	putative hydrolase	Francci3_2567	682
hypothetical protein	Francci3_0532	1022	Alcohol dehydrogenase	Francci3_1544	644	*transposase IS116/IS110*	*Francci3_2124*	*681*
acyl transferase region	Francci3_0991	1015	*hypothetical protein*	*Francci3_2440*	*642*	hypothetical protein	Francci3_1807	675
Superoxide dismutase	Francci3_2817	1013	Tetratricopeptide TPR_4	Francci3_1951	639	hypothetical protein	Francci3_1805	675
hypothetical protein	Francci3_2185	1007	**hypothetical protein**	**Francci3_0227**	**635**	hypothetical protein	Francci3_2364	675
hypothetical protein	Francci3_4343	1006	hypothetical protein	Francci3_2315	634	hypothetical protein	Francci3_2380	671
*serine/threonine kinase*	*Francci3_4051*	*989*	hypothetical protein	Francci3_4019	633	response regulator receiver	Francci3_4048	670
acyl-CoA dehydrogenase	Francci3_1000	989	hypothetical protein	Francci3_0949	633	putative O-methyltransferase	Francci3_0204	670
conserved hypothetical protein	Francci3_0096	986	serine phosphatase	Francci3_3249	632	channel protein	Francci3_3898	669
hypothetical protein	Francci3_3886	983	Amino acid adenylation	Francci3_2459	632	hypothetical protein	Francci3_2032	667
*Rhodanese-like *	*Francci3_2753*	*982*	*transposase IS116/IS110*	*Francci3_2124*	*630*	hypothetical protein	Francci3_1459	664
hypothetical protein	Francci3_4042	973	hypothetical protein	Francci3_3417	628	flavoprotein	Francci3_1816	662
**hypothetical protein**	**Francci3_3999**	**971**	**Antibiotic biosynthesis**	**Francci3_0875**	**626**	hypothetical protein	Francci3_0160	660
protein of unknown function	Francci3_2628	958	protein of unknown function	Francci3_1406	621	AMP-dependent synthetase	Francci3_1806	659
LuxR family regulator	Francci3_3253	958	hypothetical protein	Francci3_3247	621	serine/threonine protein kinase	Francci3_3395	659
50SribosomalproteinL24	Francci3_0593	944	hypothetical protein	Francci3_2943	620	hypothetical protein	Francci3_4161	655
ribosomal protein S2	Francci3_3581	936	transcription factor WhiB	Francci3_3790	618	hypC/hupF	Francci3_1946	655
hypothetical protein	Francci3_2736	934	hypothetical protein	Francci3_3997	618	hypothetical protein	Francci3_0494	655
hypothetical protein	Francci3_2269	932	transcriptional regulator	Francci3_4158	614	transcriptional regulator	Francci3_0985	654
hypothetical protein	Francci3_2809	929	hypothetical protein	Francci3_2184	610	Excisionase/Xis, DNA-binding	Francci3_1856	653
acyl-CoA dehydrogenase-like	Francci3_0053	915	hypothetical protein	Francci3_0054	608	phosphohydrolase	Francci3_1134	648
**Antibiotic biosynthesis**	**Francci3_0875**	**911**	CRISPR-associated protein	Francci3_0023	608	**SsgA**	**Francci3_3418**	**646**
2-oxoacid oxidoreductase	Francci3_3248	906	Recombinase	Francci3_2373	607	**major facilitator MFS_1**	**Francci3_2752**	**643**
translationinitiationfactorIF-1	Francci3_0605	904	*CRISPR-associated protein*	*Francci3_3345*	*606*	Inorganic diphosphatase	Francci3_4310	636
electron transfer flavoprotein	Francci3_3659	889	hypothetical protein	Francci3_2219	606	hypothetical protein	Francci3_1032	636
hypothetical protein	Francci3_4326	884	hypothetical protein	Francci3_3299	605	DNA-directed RNA polymerase	Francci3_3194	635
50SribosomalproteinL33	Francci3_0563	880	LuxR family regulator	Francci3_3253	604	**chaperoninGroEL**	**Francci3_4398**	**635**
hypothetical protein	Francci3_3625	856	*hypothetical protein*	*Francci3_2101*	*604*	**UspA**	**Francci3_2760**	**633**
Cytochrome-c oxidase	Francci3_2009	855	transcriptional regulator	Francci3_1674	600	**Aldehyde dehydrogenase**	**Francci3_2944**	**632**
GrpE protein	Francci3_4353	846	*transcriptional regulator*	*Francci3_0908*	*596*	hypothetical protein	Francci3_1014	631

^1 ^Gene annotations and locus tag numbers are colored based on their presence in all three samples (bold), in the 3dN2 and 5dNH4 samples (italic), in the 3dN2 and 3dNH4 samples (underscore), in the 3dNH4 and 5dNH4 samples (italic/underscore), and in one of the three samples (normal font).

^2 ^RPKM (Reads per Kilobase Million) = (# reads per ORF)/(size of ORF in kilobases × millions of reads in the dataset).

**Figure 1:**
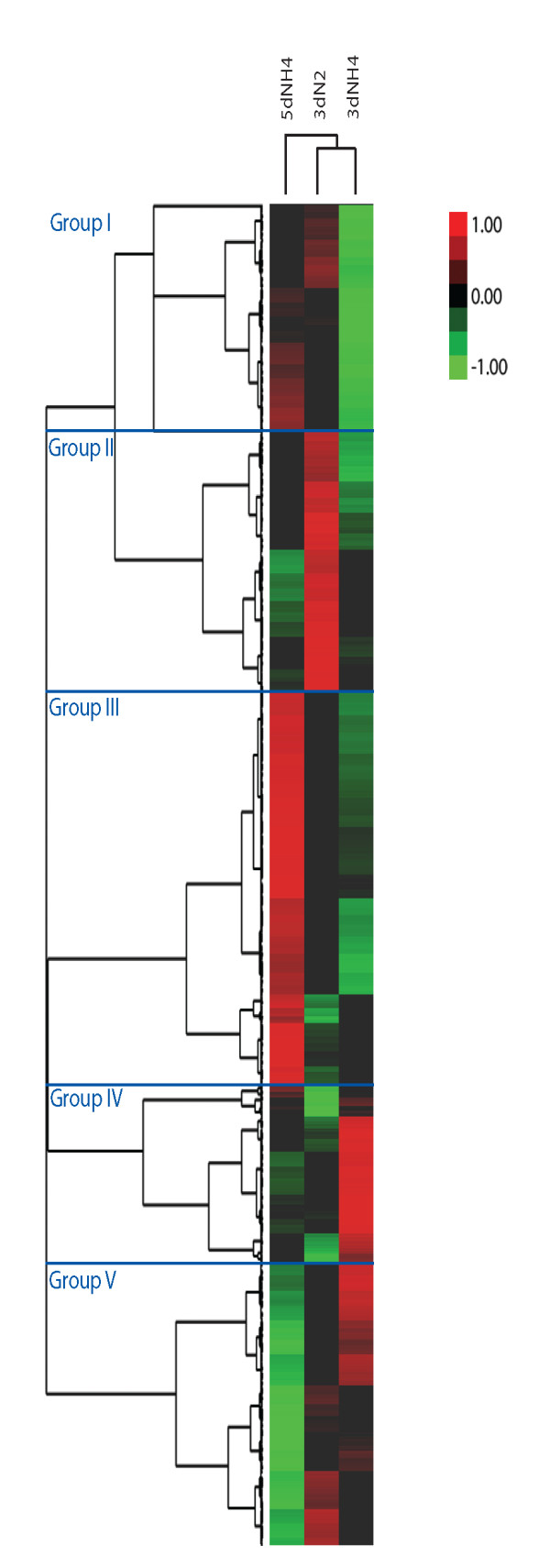
**Heat map representation of pair-wise gene expression in each sample**. The dendrogram at the top of the figure indicates relatedness of the three samples based on overall gene expression values. The dendrogram on the left side of the figure orders genes into groups based on the divergence of expression values among the three samples. The colors display gene expression variance: red indicates a higher gene expression, green indicates lower expression and black indicates the median value. This figure was generated using a log scale of RPKM values.

Comparison of the top 100 gene lists with each other (color coded in Table 2) and construction of heat maps of all genes revealed that overall gene expression varied more with culture age (three versus five days) than culture condition (+/- NH_4_^+^), with 3dNH4 and 3dN2 clustering before the 5dNH4 sample (Figure 1). Gene dendrograms (left side of the figure) gave five clusters of genes (Groups I through V) that had within-group expression profiles consistent among the three culture conditions tested. The genes in each cluster are listed in Additional File 1: Gene_list.xls.

Group I genes are clearly down-regulated in 3dNH4 cells; these include 30 transporter related genes, five diguanylate cyclases and an array of putative N-controlled proteins such as assimilatory nitrate reductase, adenosine deaminase, allantoinase and nitrogen fixation (*nif*) genes in addition to 252 hypothetical proteins. Group II genes are up regulated in 3dN2 cultures and include most of the *nif *genes, genes involved in sulfur metabolism and iron-sulfur protein synthesis, cell division proteins and hydrogenase synthesis. The 3dN2 culture was prepared with a modified iron stock containing a higher concentration of iron sulphate and sodium molybdate [24]. We cannot rule out that an increase in iron-sulfur protein synthesis may be related to the increase in iron sulphate to the medium although it is more likely to be related to an increased demand for iron and molybdenum. Eight phage integrases were also present in Group II, which was the highest number of integrases present in any of the five groups. Group III contains genes that have relatively more transcripts in 5dNH4 cells; these include a larger proportion of hypothetical protein ORFs (523 ORFs) than were present in the other four groups (average of ~200 ORFs per group). All of the annotated excisionase/Xis ORFs were present in the Group III list, suggesting that phage-related excisionases are being transcribed more in the 5dNH4 sample than in the other conditions. Group IV genes were more abundantly transcribed in the 3dNH_4_^+ ^sample including several sigma factors; this group also had the fewest transposase ORFS (2 ORFs). Group V contains ORFs more highly expressed in younger cultures. ORFs in this grouping include 17 ribosomal protein ORFs, and a majority of the glycolytic enzymes.

As expected, *nif *ORFs were more highly expressed in the 3dN2 sample, with numerous vesicles present, than in the 3dNH4 sample and were in Group II on the heat map. The 5dNH4 culture also had *nif *expression above that detected in the 3dNH4 culture. Three *nif *ORFs were not significantly expressed in the 5dNH4 sample over the 3dNH4 sample as predicted by a Kal's ztest p value [25] (Table 3). On the other hand, the genes for the core nitrogenase components nitrogenase reductase (*nifH*), and nitrogenase alpha and beta chains (*nifKD*) were upregulated in the 3dN2 sample, and were cotranscribed to similar extents within individual cultures, suggesting that they exist in an operon independent from the rest of the *nif *cluster. An intergenic space consisting of 208 nucleotides between these three ORFs and the rest of the cluster supports this analysis. The presence of *nif *transcripts in all cell types, even where ammonia should still be in excess, is in concert with the heterogeneous nature of the frankial growth habit, where mycelia develop microsites that are potentially nutrient deficient or microaerobic due to adjoining cell populations. The 5dNH4 cells are most likely depleted for combined nitrogen and, indeed, a few vesicles can be observed in older cultures. This observation highlights a fundamental problem with the mRNA deep sequencing of a *Frankia *culture where different cell physiologies can skew average gene expression in a culture. Apart from isolated vesicles [26] that are unlikely to give a sufficient quantity of mRNA for second generation sequencing technologies, long-read, single molecule sequencing techniques run in parallel could specifically sequence the transcriptome of distinct cell morphologies in a pure culture as was recently done with *Vibrio cholerae *[27].

**Table 3:** Fold changes of *nif *cluster ORF expression levels^1^

Feature ID	Annotation	5dNH4 vs 3dNH4	3dN2 vs 3dNH4	3dN2 vs 5dNH4
Francci3_4473	thiamine pyrophosphate enzyme-like TPP-binding	1.28	1.89	1.48
Francci3_4474	pyruvate flavodoxin/ferredoxin oxidoreductase-like	1.60	1.93	1.20
Francci3_4475	aminotransferase, class V	2.90	1.52	0.90
Francci3_4476	UBA/THIF-type NAD/FAD binding fold	1.20*	2.08	1.73
Francci3_4477	HesB/YadR/YfhF	2.09	2.00	0.04
Francci3_4478	nitrogenase cofactor biosynthesis protein NifB	1.35	2.17	1.61
Francci3_4479	NifZ	0.54	1.45	2.23
Francci3_4480	nitrogen fixation protein NifW	2.49	2.14	0.16*
Francci3_4481	protein of unknown function DUF683	2.81	1.75	0.61
Francci3_4482	protein of unknown function DUF269	0.23*	1.44	1.77
Francci3_4483	Dinitrogenase iron-molybdenum cofactor biosynthesis	1.82	2.03	1.12*
Francci3_4484	nitrogenase molybdenum-iron cofactor biosynthesis protein NifN	2.55	1.78	0.43
Francci3_4485	nitrogenase MoFe cofactor biosynthesis protein NifE	1.47	1.92	1.31
Francci3_4486	nitrogenase molybdenum-iron protein beta chain	1.16*	2.40	2.08
Francci3_4487	nitrogenase molybdenum-iron protein alpha chain	1.62	2.94	1.82
Francci3_4488	nitrogenase iron protein	1.34	3.71	2.77

^1^Fold changes calculated as quotients of RPKM values

* Insignificant p value as determined by Kal's ztest.

### Insertion Sequences

Recent studies on *Frankia *proteomes have indicated the presence of several transposases in CcI3 grown in culture and in symbiosis [28], raising the question of how IS elements behave in cultured CcI3 cells. Given the number of transposase ORFs in the CcI3 genome (148 complete plus 53 fragments identified by PSI-BLAST analysis [2]), mRNA deep sequencing provides an efficient method of quantifying their behavior in cultures grown under different conditions.

**Figure 2:**
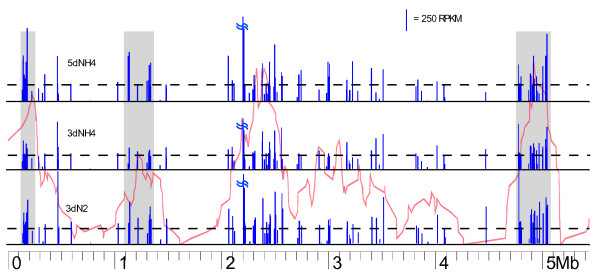
**Plot of transposase transcript RPKM values against previously determined transposase gene clusters**. Scale on the bottom represents the genome coordinates in Mb. The red line indicates the density of transposase ORFs in a 250 kb moving window in the CcI3 genome. Blue bars indicate RPKM values of each transposase ORF in the indicated growth conditions. The dotted line indicates the median RPKM value for all ORFs within the sample. Grey boxes indicate previously determined active deletion windows [3]. An IS66 transposase transcript having an RPKM value greater than 1600 in all three samples is indicated with a broken line.

**Figure 3:**
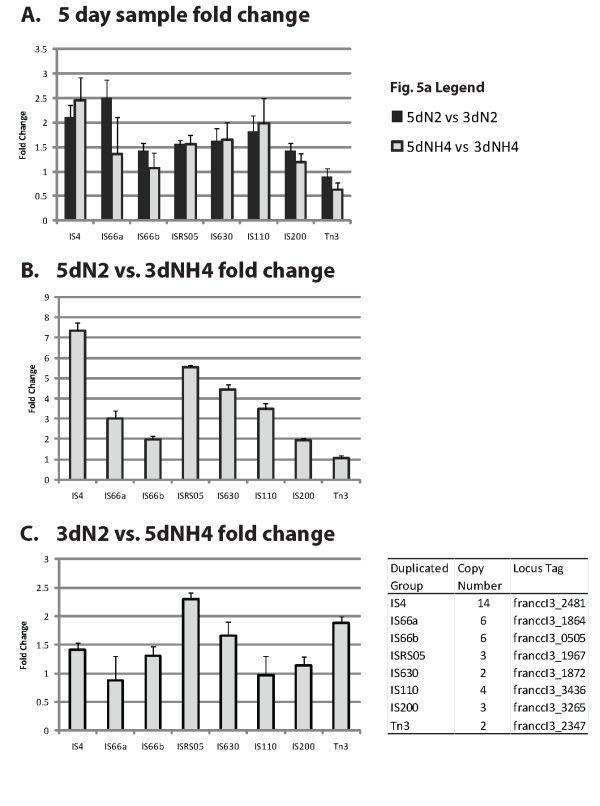
**Results of the RT-qPCR assay of highly duplicated transposase ORFs**. All values indicate relative fold increase of transcription between samples standardized against *glnA *transcript levels. Panel A - fold changes of transcripts between five day and three day time points of cultures grown on N2 (black bars) or NH4 (gray bars). Panel B: fold changes of 5dN2 vs 3dNH4. Panel C: fold changes of 3dN2 vs 5dNH4 transposase ORFs respectively. The table (inset) indicates the copy number of duplicated transposase ORFs within each IS group as well as the locus tag of one of the representative members of that group. Error bars indicate standard error of triplicate reactions over each histogram.

RPKM values for the transposase ORFs were plotted against the locations of IS elements in strain CcI3 (Figure 2; [3]). Additional files 2, 3, 4, 5, 6 and 7 list the calculated expression data for the transposase ORFs. Transposase transcripts were generally more abundant than the transcriptome's median RPKM value (dashed line; values respective of sample) throughout the genome. The visual representation of transcript abundance in Figure 2 indicates that transposase ORFs were overall more highly expressed in older cultures and, to a lesser extent, in N_2 _fixing cells than in younger, nutrient sufficient cultures. Seventy-three transposase ORFs in the 5dNH4 sample were more highly expressed with respect to the 3dNH4 sample (Figure 2; Additional file 8: SNP_call_list.xls). Only 29 transposase ORFs were shown statistically to have higher expression in 3dNH4 than in 5dNH4. A similar trend was noticed in the 3dN2 vs 3dNH4 sample, with 91 transposase ORFs having statistically significant higher expression values in the 3dN2 sample. Many transposase ORFs had similar expression in the 3dN2 vs 3dNH4 and the 5dNH4 vs 3dNH4 comparisons. This is reflected in the ztest p values, as the 3dN2 vs 3dNH4 comparison had 50 changes with p values greater than 0.05 and the 5dNH4 versus 3dNH4 comparison had 48 changes with p values greater than 0.05. The majority of the insignificant p values in the comparisons are due to similarity of RPKM values.

One IS66 transposase (Locus tag: Francci3_1864) near the 2 Mb region of the genome had an RPKM greater than 1600 in all samples. The majority of these reads were ambiguous. This transposase has five paralogs with greater than 99% nucleotide similarity, thereby accounting for ambiguous reads, so the elevated RPKM, while still high, is distributed among several paralogs. Other transposase ORFs with RPMK values higher than the median were more likely to be present in CcI3 deletion windows (gray boxes [3]) as determined by a Chi Square test against the likelihood that high RPKM transposase ORFs would exist in a similar sized region of the genome at random (p value = 1.32 × 10^-7^). This observation suggests that any transposase found in these windows is more likely to be transcribed at higher levels than transposases outside of these regions.

The largest change in expression was found in an IS3/IS911 ORF between the 5dNH4 and 3dNH4 samples. This ORF (locus tag: Francci3_1726, near 1.12 Mb) was expressed eleven fold higher in the 5dNH4 sample than in the 3dNH4 sample. Five other IS66 ORFs are also highly expressed in 5dNH4 ranging from 4 fold to 5 fold higher expression than in the 3dNH4 sample. Eight IS4 transposases had no detected reads under the alignment conditions in each growth condition. These eight IS4 transposases are members of a previously described group of 14 paralogs that have nearly 99% similarity in nucleic acid sequence [3]. Parameters of the sequence alignment used allowed for ten sites of ambiguity, therefore discarding reads from eight of these 14 duplicates as too ambiguous to map on the reference genome. Graphic depictions of assembled reads derived from raw CLC workbench files show that the majority of reads for the six detected IS4 transposases mapped around two regions. Both of these regions contained one nucleotide difference from the other eight identical transposases. *De novo *alignment of the unmapped reads from each sample resulted in a full map of the highly duplicated IS4 transposase ORFs (data not shown).

More globally, the 5dNH4 and 3dN2 samples had higher RPKM values per transposase ORF than in the 3dNH4 sample. The sum of the RPKM values among the transposase data set placed the 5dNH4 sample (34350 sum RPKM) and the 3dN2 (36150 sum RPKM) each nearly 30% higher than in 3dNH4 (26916 sum RPKM). The numbers of transposase genes classified as upregulated in the heat maps in Figure 1 include 44 in 3dN2 cells, 40 in 5dNH4 cells and only two in 3dNH4 cells. Twenty-eight were down regulated in the 3dNH4 cells as shown by the heat map analysis (Additional File 8: SNP_call_list.xls). These results suggest a relative quiescence of transposase ORFs during healthy growth, and a burst of transcription when cells are stressed. Mutagenesis of genes involved in general metabolic pathways in *Escherichia coli *has been shown to promote earlier transposition of an IS5 family insertion sequence [29]. Media supplements to the mutated cells were shown to delay transposition events, thereby showing general starvation responses were likely involved in increased IS element activity [29].

The expression of *nif *cluster genes in the 5dNH4 sample suggests that the ammonium content of the medium was depleted, or nutrient deprived microsites had developed among the mycelia. One of the highly expressed non-ribosomal ORFs is the pyrophosphohydrolase gene *hisE *(Francci3_4317), suggesting that the amino acid histidine is in short supply. Additionally, a serine O-acetyltransferase was highly expressed in 5dNH4 cells, indicating activity in the cysteine synthesis pathway. Higher expression of both *ppx/gppA *ORFs (Locus tags: Francci3_0472 and Francci3_3920) in the 5dNH4 sample suggests that the stringent response [30] is active in response to amino acid deprivation. Two ORFs annotated as (p)ppGpp synthetases (Locus tags: Francci3_1376 and Francci3_1377) were actually more highly expressed in 3dN2 and 3dNH4 cells than in 5dNH4 cells.

Transcription of IS elements does not directly correlate to translation [31]. Many IS elements prevent their own transposition by requiring a -1 frame shift mutation in the transcript in order to express a functional transposase protein [32]. Since the specific methods of translational control used by *Frankia *IS elements are unknown, transcriptome data alone cannot be used as a proportional metric for transposition activity. On the other hand, recent proteomic studies on the CcI3 genome have confirmed that translation of many IS elements does occur *in vivo *and in symbiosis [16,33].

### RT-qPCR confirmation of transposase transcription

Duplicated copies of highly similar transposase ORFs presented a problem in the analysis of transcript sequence data. To compare transcription frequencies of duplicated ORFs in different culture conditions, we used RT-qPCR to amplify conserved regions of eight duplicated transposase ORF families using primers designed to amplify conserved regions in each group. The duplicates had greater than 98% nucleotide similarity with each other. The glutamine synthetase I (*glnA*) gene was used to normalize expression data as previously described [34]. We included a five-day old nitrogen fixing (5dN2) condition in our assay to better estimate transposase ORF expression in two older culture conditions (5dN2 and 5dNH4).

The results of the RT-qPCR assay confirmed the transcriptome sequence data (Figure 3). Comparing the five-day samples with three-day samples revealed an increase in transposase ORF transcription in older cultures in nearly all cases (Figure 3a). The only exception was in the case of the Tn3 family of transposases where transcription was predicted to be higher (fold change values less than one) at three days in both conditions. This may be due to transposition immunity described for other members of the Tn3 family [35]. Cross comparisons of NH4 and N2 samples revealed that nitrogen fixing cultures had more transposase transcripts from these duplicated families than from the ammonium cultures at both time points (Figures 3b and 3c). The most dramatic change in transcript quantity was found for the IS4 transposases' transcripts in the 5dN2 sample that were 7.4 fold higher than levels in the 3dNH4 sample. As the representative transposase ORFs chosen for the RT-qPCR analysis were families of duplicates, a direct comparison of RT-qPCR fold change to transcriptome RPKM values was difficult to make. Still, the results of this experiment confirm the general trend of transposase ORF transcription in *Frankia *sp. CcI3: older and nitrogen-deprived cultures had higher transcription of transposase ORFs.

### Prophage and CRISPRs

ORFs with phage-related annotations were all more highly transcribed in the five-day sample with respect to both three-day samples (Table 4). Several ORFs annotated as phage integrases were expressed more than two-fold in the 5dNH4 sample when compared to the 3dNH4 sample. Comparisons of fold change among all three samples yielded many statistically insignificant differences as determined by a Kal's z-test suggesting that these ORFs are likely transcribed at similar rates regardless of culture conditions. A phage SPO1 DNA polymerase-related protein (Francci3_0075) was constitutively expressed in all three samples, and four phage resistance ORFs were up-regulated in the 5dNH4 sample. The latter include members of the *pspA *and *pgl *(Phi C31) families of phage resistance genes. Similar RPKM values between the two *pgl *ORFs in all three samples suggest that these ORFs are transcribed as an operon in CcI3.

**Table 4:** Fold changes of phage related ORFs^1^

Feature ID	Annotation	5dNH4 vs 3dNH4	3dN2 vs 3dNH4	3dN2 vs 5dNH4
Francci3_0075	phage SPO1 DNA polymerase-related protein	-1.02*	1.19*	1.21*
Francci3_0114	phage integrase	-1.10*	1.54	1.70
Francci3_0407	phage integrase	1.48	1.23	-1.20
Francci3_0878	phage integrase	1.05*	1.55	1.48
Francci3_1095	phage integrase	1.46	1.62	1.11
Francci3_1144	phage integrase	2.72	1.63	-1.67
Francci3_1203	phage integrase	1.39	1.66	1.20
Francci3_1870	phage integrase-like SAM-like	3.05	1.53	-2.00
Francci3_2053	phage integrase-like SAM-like	-1.32	1.83	2.43
Francci3_2147	phage integrase	1.92	1.52	-1.26
Francci3_2228	phage shock protein A, PspA	2.47	1.43	-1.73
Francci3_2304	phage integrase	1.60	-1.24*	-1.99
Francci3_2344	phage integrase	1.59	1.20*	-1.32
Francci3_2443	putative phage-related terminase large subunit	1.34	1.84	1.37
Francci3_2954	bacteriophage (phiC31) resistance gene PglY	1.57	1.38	-1.14*
Francci3_2955	bacteriophage (phiC31) resistance gene PglZ	1.47	1.22*	-1.21*
Francci3_3052	phage integrase	1.07*	1.43	1.34
Francci3_3350	phage integrase	1.42	1.74	1.22
Francci3_3388	phage integrase	1.55	1.84	1.19
Francci3_3390	phage integrase	1.89	-1.09*	1.73
Francci3_3532	phage integrase	2.02	1.48	-1.36
Francci3_3535	phage shock protein A, PspA	-1.98	-1.86	1.06*
Francci3_3583	phage integrase	-1.34	1.39	1.86
Francci3_3734	phage integrase-like SAM-like	1.34	1.62	1.21
Francci3_4274	phage integrase	4.52	1.60	-2.83
Francci3_4338	phage integrase	-1.36	1.69	2.30

^1^Fold changes calculated as quotients of RPKM values

*Insignificant p value as determined by Kal's ztest.

Negative values indicate a fold reduction of expression in the reference (later) condition.

CcI3 has four putative CRISPR arrays, two of which are located near clusters of CAS ORFs (data obtained from CRISPRFinder [36]). Three of the CRISPR arrays had high numbers of repeat copies (38, 15 and 20 spacers per array ordered with respect to the OriC) making alignment of ambiguous sequence reads difficult. Even the shorter 36 bp read lengths of the 5dNH4 sample could not be reliably mapped across the arrays using the CLC Genome Workshop alignment programs. As a result, few reads mapped to the array region of the CRISPR islands and numerous deletions were predicted (Additional Files 2 through 7). The CAS ORF transcripts, by contrast, were detected in all three samples. Again, transcription was modestly higher in the 5dNH4 sample than in the 3dNH4 sample (Table 5). In this instance, the 3dN2 sample had nearly two fold higher expression of all CAS ORFs when compared with the 3dNH4 sample. Comparison of the 5dNH4 and 3dN2 samples revealed insignificant fold changes as determined by a Kal's ztest.

**Table 5:** Fold changes of CRISPR associated ORFs^1^

Feature ID	Annotation	5dNH4 vs 3dNH4	3dN2 vs 3dNH4	3dN2 vs 5dNH4
Francci3_0017	CRISPR-associated helicase Cas3, core	1.31	1.39	1.06*
Francci3_0020	CRISPR-associated protein, CT1975	2.99	1.63	-1.84
Francci3_0021	CRISPR-associated protein, CT1976	2.79	1.42	-1.96
Francci3_0023	CRISPR-associated protein Cas1	1.31	1.57	1.20
Francci3_0024	CRISPR-associated protein, Cas2	1.16	1.31	1.13*
Francci3_3341	CRISPR-associated helicase Cas3, core	1.29	1.35	1.05*
Francci3_3344	CRISPR-associated protein TM1801	1.04*	1.45	1.39
Francci3_3345	CRISPR-associated protein Cas4	1.97	1.36	-1.44
Francci3_3346	CRISPR-associated protein Cas1	1.14	1.29	1.13

^1^Fold changes calculated as quotients of RPKM values

*Insignificant p value as determined by Kal's ztest.

Negative values indicate a fold reduction of expression in the reference (later) condition.

### SNP detection

Given the base pair resolution of RNA sequencing, it is possible to identify single nucleotide polymorphisms (SNPs). Recent analysis of the bovine milk transcriptome revealed high fidelity of SNP calls derived from an RNA-seq experiment, though the authors caution that stringent criteria are necessary to reduce false positive calls [37]. Using similar filtering criteria, we identified 215 SNPs in the 5dNH4 sample, 365 SNPs in the 3dN2 sample and 350 SNPs in the 3dNH4 sample. Comparison of the SNP populations revealed that the 5dNH4 sample had substantially different SNP calls than the 3dN2 and 3dNH4 samples. Only 21 of the putative SNPs were found in all three samples (Table 6). Twelve of these common SNPs resulted in non-synonymous amino acid changes.

**Table 6:** Detected SNPs present in all three samples

Locus tag	Annotation	Position	**Reference**^**1**^	**Variants**^**2**^	Amino Acid Change
Francci3_0398	putative DNA-binding protein	452	G	G/A	Arg -> Gln
Francci3_1612	NLP/P60	356	G	G/A	Arg -> Gln
		375	A	A/C	Gln -> His
Francci3_1959	Transposase, IS110	1109	G	G/A	Gly -> Asp
Francci3_2025	Transposase, IS4	81	G	A/G	-
		91	C	C/T	Arg -> Cys
		119	T	T/C	Val -> Ala
Francci3_2063	hypothetical	310	A	A/C	Met -> Leu
		313	C	C/T	Pro -> Ser
		333	C	C/T	-
		353	A	A/G	Glu -> Gly
Francci3_3047	Radical SAM	93	G	G/C	-
Francci3_3251	putative signal transduction histidine kinase	293	T	C/T	Val -> Ala
Francci3_3418	SsgA	165	C	T/C	-
Francci3_4082	dnaE	3579	T	C/T	-
		3601	G	G/A	Glu -> Lys
Francci3_4107	Integrase	135	C	C/T	-
Francci3_4124	Recombinase	162	T	T/A	-
		168	C	T/C	-
Francci3_4157	Hypothetical	36	C	C/T	-
		49	A	A/G	Ser -> Gly

^1 ^The nucleotide present in the reference genome sequence of *Frankia *sp. CcI3.

^2 ^The predicted allelic variants for the reference position nucleotide. The most common polymorphic nucleotide is listed first in the proportion.

There are several possibilities that may explain the variance of SNP content between the 5dNH4 sample and the two three day samples. The age of the culture is a possible, yet unlikely, contributor to a significantly different SNP pattern. *Frankia *strains are maintained by bulk transfer of cells since derivation from single colonies is problematical due to the hyphal habit of growth. Thus, over time, SNPs likely arise spontaneously. Another possibility is that errors are incorporated into the mRNA-seq libraries resulting in false positive SNPs. The Superscript III^© ^reverse transcriptase used in the first strand cDNA synthesis was derived from a MML virus [38] and has an error rate of approximately 3.0 × 10^-5 ^errors per base [39]. Therefore, only SNPs detected in all three samples with high coverage and multiple variant copies were likely true positive SNPs.

## Conclusions

We deep-sequenced dscDNA libraries derived from three culture conditions of *Frankia *sp. CcI3. Overall gene expression varied more as a function of culture age than as a function of nitrogen deprivation, likely because the cell population has fewer actively growing cells at the fifth day of culture and those remaining are adapting to nutrient deprivation. In two limited nutrient environments, transposase ORFs were relatively more highly expressed than in younger ammonium grown cells. A RT-qPCR assay designed to quantify highly duplicated transposase ORFs supported the data from the mRNA-seq experiment. These results, in tandem with discovery of putative SNPs, suggests that the IS element laden CcI3 genome is in constant flux within the relatively mundane conditions of a culture flask.

## Methods

### Culture media and conditions

Frozen stocks of *Frankia *sp. strain CcI3, were suspended in duplicate in 200 ml of *Frankia *Defined Minimal media (FDM) containing 45 mM sodium pyruvate and 9.3 mM ammonium chloride in 500 ml flasks [40]. Cells were grown at 30°C for three or five days on FDM with or without (N_2 _fixing cells) ammonium. Nitrogen fixing cultures were prepared using a modified iron stock as previously described [24]. Given the difficulty in quantifying viable *Frankia *cells in culture, a total of three ml of gravity-settled cells were harvested per culture flask for RNA extraction.

### RNA extraction

*Frankia *cells were processed using a ZR Fungal/Bacterial RNA MiniPrep™ kit from Zymo Research^© ^(http://www.zymoresearch.com) using the manufacturer's recommendations. To completely remove genomic DNA (gDNA) contamination from the RNA extraction, we performed the in-column DNAse I optional step using Amplification grade DNAse I (Invitrogen™, http://www.invitrogen.com). DNAseI incubation times were extended to 30 minutes at 37°C in order to completely remove gDNA from the sample. A final elution volume of 15 μl of RNAse free water was used instead of the recommended 6 μl elution volume. Only RNA samples with a 260/280 nm wavelength ratio above 2.00 were used for library construction and RT-qPCR assays.

In order to enrich mRNA content for generating a cDNA library, we used the MICROBExpress™ Bacterial mRNA Enrichment Kit (Ambion Inc., http://www.ambion.com). The manufacturer's website specifies that the oligonucleotide sequence used by the kit should anneal to the 16S and 23S rRNA sequences of many eubacterial species including *Frankia *sp. Approximately 10 μg of *Frankia *total RNA in each condition was processed using the kit per the manufacturer's instructions. This procedure yielded 2 - 3.75 μg of RNA after depletion for each sample. Subsequent gel analysis and sequencing data revealed substantial 16S and 23S rRNA within the sample, suggesting only partial depletion of rRNA transcripts. Samples were nonetheless prepared using the depletion kit in order to minimize variability due to differential handling in the experiment.

### Complementary DNA library generation

One microgram of processed *Frankia *RNA was used in an Illumina mRNA-seq kit. The poly-dT pulldown of polyadenylated transcripts was omitted, and the protocol was followed beginning with the mRNA fragmentation step. A SuperscriptIII^© ^reverse transcriptase was used instead of the recommended SuperscriptII^© ^reverse transcriptase (Invitrogen™). This substitution was made in light of the higher G+C% of *Frankia *sp. transcripts (71% mol G+C) and the ability of the SuperscriptIII^© ^transcriptase to function at temperatures greater than 45°C. Because of this substitution, the first strand cDNA synthesis stage of the protocol could be conducted at 50°C instead of 42°C. Since a second-strand cDNA synthesis was performed, the cDNA library was agnostic with respect to the strandedness of the initial mRNA. The final library volumes were 30 μl at concentrations of 40 - 80 ng/μl as determined by Nanodrop spectrophotometer.

### Library clustering and Illumina platform sequencing

Prior to cluster generation, cDNA libraries were analyzed using an Agilent^© ^2100 Bioanalyzer (http://www.chem.agilent.com) to determine final fragment size and sample concentration. The peak fragment size was determined to be approximately 200 +/- 25 bp in length for each sample. Twenty nmoles of each cDNA library were prepared using a cluster generation kit provided by Illumina Inc. The single-read cluster generation protocol was followed. Final cluster concentrations were estimated at 100,000 clusters per tile for the five day sample and 250,000 clusters per tile for the two three day samples on each respective lane of the sequencing flow-cell.

An Illumina^® ^Genome Analyzer IIx™ was used in tandem with reagents from the SBS Sequencing kit v. 3 in order to sequence the cDNA clusters. A single end, 35 bp internal primer sequencing run was performed as per instructions provided by Illumina^®^. Raw sequence data was internally processed into FASTQ format files which were then assembled against the *Frankia *sp. CcI3 genome [Genbank: CP000249] using the CLC Genomics Workbench™ software package distributed by CLC Bio^©^.

*Frankia *sp. CcI3 has a several gene duplicates. This made the alignment of the short reads corresponding to the gene duplicates difficult. Reads could only be mapped to highly duplicated ORFs by setting alignment conditions to allow for 10 ambiguous map sites for each read. In the case of a best hit "tie," an ambiguous read was mapped to a duplicated location at random. Without this setting, more than 20 ORFs would not have been detected by the alignment program simply due to nucleotide sequence similarity.

To standardize gene expression calculations among different samples, the CLC Genomic Workbench software calculates an expression value termed "reads per kilobase million" (RPKM). This calculation incorporates variable gene length in the gene expression ratio, and the total number of reads obtained from a sequencing run [41]. The equation used to determine RPKM values is as follows:

$$RPKM=NumberofReads∕\left(Kilobaselengthofgene*Millionsofreadsindataset\right)$$

The RPKM value allows comparisons between datasets containing variable numbers of reads as well as expression of genes with varying lengths. Because of the disparate quantities of rRNA reads among the three samples, we removed all non-coding RNA (ncRNA) reads from the data set before calculating RPKM values. This ensures that the reads from the 5dNH4 sample, which had the lowest number of ncRNA reads, were not overrepresented. Comparisons of gene expression were tested using Kal's Z-test [25]. Heat maps were generated using the Cluster 3.0 command line program (http://bonsai.ims.u-tokyo.ac.jp/~mdehoon/software/cluster/software.htm). Datasets were normalized and median subtracted prior to map generation. Maps were viewed using Java Treeview [42].

Potential SNPs were filtered using the following criteria: (1) reads containing putative SNPs were discarded if they had an average quality score of less than 15; (2) the polymorphic base within the read had to have a quality score above 20; (3) at least 10× coverage of the SNP position was required; (4) the SNP had to be present in 25% of the reads at that location. Raw sequence reads and calculated RPKM values for each CcI3 ORF were uploaded to the Gene Expression Omnibus database at NCBI (http://www.ncbi.nlm.nih.gov/projects/geo) with the accession number GSE30680.

### RT-qPCR assays

The nucleotide sequences for the target transposase ORFs in *Frankia *strain CcI3 [genbank: CP000249] were retrieved from Genbank. Primers were designed using the Primer3 webtool (http://frodo.wi.mit.edu/primer3/) with settings to generate primers with a melting temperature of ~60°C. Due to the limitations of extension time in quantitative polymerase chain reactions (qPCR), primers were designed to amplify less than 200 bp of sequence when possible.

Stocks of *Frankia *sp. CcI3 cells were grown in four culture conditions that included two time points and two medium types. Three of the conditions mirrored those used in the mRNA-seq experiment (3dN2, 3dNH4 and 5dNH4). A fourth condition, consisting of cells grown in nitrogen fixing medium for five days (5dN2), was also used. Cells were harvested and RNA was purified in the same manner as used in the mRNA-seq experiment. Approximately one micro-gram of RNA from each sample was used in subsequent reverse transcriptase reactions. Complementary DNA was synthesized using the SuperscriptIII^© ^reverse transcriptase with gene specific primers (~100 nM final concentrations per reaction mix). Synthesis of the first strand was carried out at 55°C for 50 minutes with a five minute denature step at 80°C. RT reactions were diluted ten-fold with sterile water after denaturation.

All qPCR experiments were performed using the Bio-Rad™ SsoFast^© ^Evagreen qPCR 2X master mix. Reaction volumes were reduced to 12.5 μl. A Bio-Rad™ iQ5 real-time thermocycler was used to quantify reactions. Antibody denaturing of the SsoFast polymerase was performed at 95°C for 1.5 minutes immediately prior to any cycling step. This was followed by one 98°C denaturation for 2 minutes. Temperature cycling consisted of the following: 35 cycles of 98°C for 10 seconds then 55°C for 15 seconds and finally 65°C for 15 seconds. Melt curves (to determine if there were multiple PCR amplicons) were constructed by heating final amplified reactions from 65°C to 95°C for 10 seconds in single degree stepwise fashion. Primer efficiencies were calculated from readings derived from a standard curve of known DNA concentrations. Relative expression levels of target genes were calculated using the Pfaffl standardization as previously described [34]. The glutamine synthetase I gene (*glnA*) was used as a reference gene to standardize relative expression in the four samples.

## Authors' contributions

DMB created the RNA-seq libraries. DMB and DRB planned the experiments, analyzed the data and wrote the manuscript. Both authors have read and approved of the final manuscript

## Supplementary Material

Additional file 1 **Gene lists for heatmap clusters**. List of ORFs segregated as clusters from the heat map figure (Figure 1). Click here for file

Additional file 2 **3dN2 sample dataset statistics**. Tabular output of CLC Genome Workbench software for the 3dN2 sample. Click here for file

Additional file 3 **3dNH4 sample dataset statistics**. Tabular output of CLC Genome Workbench software for the 3dNH4 sample. Click here for file

Additional file 4 **5dNH4 sample dataset statistics**. Tabular output of CLC Genome Workbench software for the 5dNH4 sample. Click here for file

Additional file 5 **Pairwise comparison of three day samples**. Comparison of RPKM values from the 3dNH4 and 3dN2 samples for annotated *Frankia *sp. strain CcI3 ORFs. Click here for file

Additional file 6 **Pairwise comparison of 3dN2 with 5dNH4**. Comparison of RPKM values from the 5dNH4 and 3dN2 samples for annotated *Frankia *sp. strain CcI3 ORFs. Click here for file

Additional file 7 **Pairwise comparison of the two NH4 grown cells**. Comparison of RPKM values from the 3dNH4 and 5dNH4 samples for annotated *Frankia *sp. strain CcI3 ORFs. Click here for file

Additional file 8 **SNP calling and filtering datasets**. Excel worksheets containing raw SNP calling data from all three RNA-seq experiments. Click here for file
